# Comparative Vitality of the Sexes

**Published:** 1896-03

**Authors:** 


### Comparative Vitality of the Sexes.

It is the common impression that men are not only less subject to illness but are longer lived than women. The life tables of insurance companies, however, show that the term of life of women is slightly longer than that of men. The difference in the mortality rates during the first few years of life is striking. During the first year the mortality among males is decidedly greater than among females. Although more boys are born than girls, the proportions are reduced to almost even terms at the end of the first year by the excessive male mortality. Even during the first four years the mortality among males exceeds that among females, notwithstanding the fact that there are practically no distinctions made in the management of the two sexes. Both are subject to the same conditions, are dressed virtually the same, and receive the same food. At about five years the comparative death-rate among girls begins to increase. This has been attributed to the fact that boys of this age are more in the open air. The mortality in both sexes diminishes from this time until the twelfth year, when it attains its lowest point. It then steadily rises, being larger in each successive year. Between the twelfth and sixteenth years the death-rate among girls increases more rapidly than among boys, but after the sixteenth year, for several years, the rate of increase is more rapid on the male side. The explanations that have been offered for these peculiarities are not wholly satisfactory; but one fact is clear, that during early years, females possess a greater tenacity of life than do males.—*Maryland Medical Journal.*

